# The Local Food Environment and Food Security: The Health Behavior Role of Social Capital

**DOI:** 10.3390/ijerph16245045

**Published:** 2019-12-11

**Authors:** Christopher J. Paul, John E. Paul, Rosa S. Anderson

**Affiliations:** 1 Department of Public Administration, North Carolina Central University, 1801 Fayetteville St., Durham, NC 27707, USA; 2 Department of Health Policy and Management, University of North Carolina at Chapel Hill, Chapel Hill, NC 27599-7400, USA; paulj@email.unc.edu; 3 College of Behavioral and Social Sciences, North Carolina Central University, Durham, NC 27707, USA; rand@nccu.edu

**Keywords:** social capital, food security, health behavior

## Abstract

Food politics and economic forces may determine the macro conditions for food supply, but the local environment has the most substantial impact on population health. Food security is determined not only by the basic availability of food, but also by social, economic, and cultural factors influencing dietary behaviors. This paper investigates the role of social institutions, specifically social capital, in affecting food security by proposing a theoretical linkage between social capital and health behavior, and an illustrative case is provided. Social capital, defined as the value of the bonding, bridging, and linking relationships between people, is well demonstrated to be related to health. Many mechanisms link social capital to health, including shared access to food and nutritional behaviors. Further, social capital influences health through social status and race. This paper further investigates the links between minority status, food security, social capital, and health. The analysis draws on empirical work in North Carolina with community gardens, faith communities, the local food environment, and other social capital-related variables. By investigating the nature of local food security, particularly for minority populations, this analysis allows for better integration of local conditions with global food politics.

## 1. Introduction

Food politics and economic forces may determine the macro conditions for the food supply [1], but it is the local environment that likely has the most substantial impact on health [2]. Food security is determined not only by the basic availability of food supply but also by social institutions, economic forces, and cultural factors influencing dietary behaviors. One social factor, social capital (the value of the bonding, bridging, and linking relationships between people), is well demonstrated to be related to health and offers many potential pathways influencing food security, including ad hoc support from community members and other informal insurance, social norms, and shared behaviors [3]. 

This paper investigates the role of social institutions, specifically social capital, in affecting food security through health behaviors. Specifically, this paper asks the question: how could greater social capital significantly improve food security? The paper first identifies theoretical relationships between food security and social capital using a health behavior intervention framework [4], and then it provides an initial investigation through an illustrative case study of these in the suburban setting of Durham, North Carolina, USA. 

Social capital has long been demonstrated to be related to health [5]. Many behavioral mechanisms link social capital to health, including access to food and good nutrition [6]. Further, social capital influences health impacts through social status and race [7,8]. Food and nutrition are major components of health and, thus, important to investigate when considering health disparities [9]. Indeed, diet and the resultant health outcomes are highly influenced by behavioral factors including geography, peer behavior, cultural factors, current health status, access to health care, and food security [10]. Socioeconomic status, minority status, and race or ethnicity also are significant factors in dietary behavior [11]. Social capital is important to health, both directly and indirectly, as social cohesion can lead to healthier behaviors as well as providing better access to resources and support for healthier lifestyles [12]. While social capital is one of many mediating factors in food security and health, evidence suggests it is a meaningful intervening variable in health and nutrition. Social capital is a particularly useful framework when considering health disparities among communities because it links individual and community characteristics.

Research in social capital varies greatly in methods and strengths of findings, but the concept has persisted across time and disciplines not because it is well accepted as a phenomenon, but because understanding the value of human relationships is at the core of social science. There has been little research considering the broad conceptions of food security in relation to social capital. This paper initially establishes theoretical relationships and mechanisms between food security, social capital, and health behavior. The arguments of the paper are in the context of a case study of a grassroots faith-based organization in Durham, North Carolina. The paper concludes by suggesting approaches for studying food security and social capital in this setting as well as the possibilities of community-based interventions with a social capital component to improve health outcomes. 

## 2. Background and Theory 

Food security is essential for leading a productive and healthy life from childhood through old age. Food insecurity, or having inadequate food, in the United States affects minority and older populations most severely. While approximately 11% (15 million) of households nationwide experience some form of food insecurity, 18% of Latino households and 22% of African American households have insufficient access to food [13].

Geography is an important factor in food security. In rural areas, availability is a greater concern, while in urban areas, cost is often the primary concern [14]. Rural households are more likely than urban households to be food insecure in the United States [15]. Substantial attention has been directed to the absences of grocery stores in neighborhoods, so called “food deserts”. The reality of food deserts is more complex, however, as availability of food stores appears inadequate to address food insecurity [16].

While macro-level forces and individual idiosyncratic factors play a role, many of the variables driving food insecurity have important social components. Specifically, the value and strength of social relationships in the form of “social capital” can inform the conditions and opportunities to improve food security. Higher social capital measures are hypothesized here to be correlated with improved food security. This relationship is predicted to be generally stable regardless of socioeconomic setting or national economic status. Further, community organizations that are associated with social capital, specifically local nonprofits and religious communities, leverage social capital to increase food security. As one particular space of social capital, religious centers, such as churches, could play an important role in improving food security. 

### 2.1. Defining Food Security and Social Capital

Food security can be more formally defined as physical, social, and economic access to sufficient, safe, and nutritious food that meets dietary needs and food preferences for an active and healthy lifestyle [17]. This definition includes not just physical, social, and economic dimensions but also the preferences and lifestyles of the individuals. The World Summit on Food Security further enumerated four pillars of food security: (1) availability, (2) access, (3) utilization, and (4) stability [18]. 

Food is at the heart of many social relationships and interactions. Social scientists need to understand the factors these relationships play in well-being for individuals and communities. Spaces where people obtain and share food are where they develop much of the “goodwill, fellowship, mutual sympathy, and social intercourse” [19] that comprise growing social capital. 

Social capital is broadly the value of social relationships. As all humans have experiences, “family, friends, and associates constitute an important asset, one that can be called upon in a crisis, enjoyed for its own sake, and/or leveraged for material gain” [20]. The value of social capital can be assessed through the “networks, norms, and social trust that facilitate coordination and cooperation for mutual benefit” [21]. Thus, social capital plays critical roles in the connection of people, the sharing of practices, and the nature of mutual cooperation, all characteristics that have substantial impacts on food security.

The social capital of relationships, whether between individuals or groups, and with varying degrees of proximity, is specifically considered here as the levels of bonding, bridging, and linking between people [3]. Bonding social capital is the strength of individual close relationships, notably family relationships. Bonding social capital is driven by “homophily”, the desire to affiliate with people of similar backgrounds [22]. Bonding social capital is linked to positive social outcomes [23]. Bridging social capital is the accumulation of “weak ties” that stretch further across heterogeneous communities to increase access to resources and opportunities [24]. The resources of these weak ties could be essential in ensuring food security. Linking social capital is the vertical connection between individuals and their communities and leaders, or positions at other levels of hierarchies. This type of social capital is essential for bringing higher-level resources back to communities. 

Each of these levels can affect food security, as discussed in the next section, directly and indirectly in the context of food systems [25]. The effect of social capital on food security may also be at the margin. Social relationships do not necessarily, or even likely, form the basis of someone’s primary food supply but instead form during particular events or periods of limited food. As with many social capital-linked phenomena, it is important to understand how social capital mediates the outcomes for both individuals and the social systems they inhabit.

### 2.2. The Role of Social Capital in Food Security

Social capital is a mediating variable for food security. This means that the effect of the variables that influence food security, such as income, education, and geography, can be altered, and usually improved, by increased social capital. The four Food and Agricultural Organization (FAO) components of food security and their intersection with social capital are discussed below and summarized through examples in Table 1. The matrix of Table 1 represents examples of the roles that each level of social capital plays in each dimension of food security.

Availability is the physical availability of food. On the macro level, this is the overall food supply determined by production, distribution, and trade. While this component focuses on the macro level, these processes are also relevant to food security on the individual level when considering activities such as household production and savings mechanisms, whether specifically of food products or of pathways for resilience. Social capital influences availability through the shared mechanisms by which people produce and store food [26]. In agrarian communities, this includes seed sharing and shared food processing centers (e.g., mills and roasters), as well as shared knowledge and predictions of risks. In urban communities, this can include food storage such as community food pantries.

Access describes economic and physical access to food, including financial ability to purchase adequate food and mobility to access it. Given that the food-insecure population generally has very limited financial resources or financial capital, the substitution of social capital may be an important path for food resources [27]. Social capital can directly serve as a form of insurance, such that food or resources to obtain food are transferred directly between networks of people [28]. Further, social capital can also facilitate solutions to geographic barriers to getting food, whether that be from shared transportation, meal delivery, or meals at community organizations and locations. Recent evidence in rural eastern North Carolina suggests that lower travel time to food sources is a key factor in food security [29].

Utilization in food security terms is the effect of the nutritional content of the food consumed. Social capital has substantial impact on this domain because nutritional behaviors are greatly influenced by peers (bonding relationships), as people model the eating behavior of those around them [30]. Socialization through bridging relationships develops shared conditions of dietary norms and characteristics, such that socioeconomic class predicts nutritional outcomes [31]. At the broadest level, linking relationships allows for dissemination of dietary information and the co-creation of programs for food security to improve food and nutritional quality [32]. 

Stability is food security across time. For those with limited capital, shocks such as interruption in income or a poorer than expected harvest in a home garden can lead to disruptions of the food supply, affecting all in the proximate bonding relationships of the local community [33]. Social capital, however, is extremely important in ensuring stability of food security, as bridging social relationships increases flexibility and informal forms of support or insurance to obtain food [34]. Access to transportation, community meals such as church potlucks, and school lunch programs all provide mechanisms for stability of food security. Linking social capital promotes stability through policies and interventions important to the community [35].

**Figure 1:**
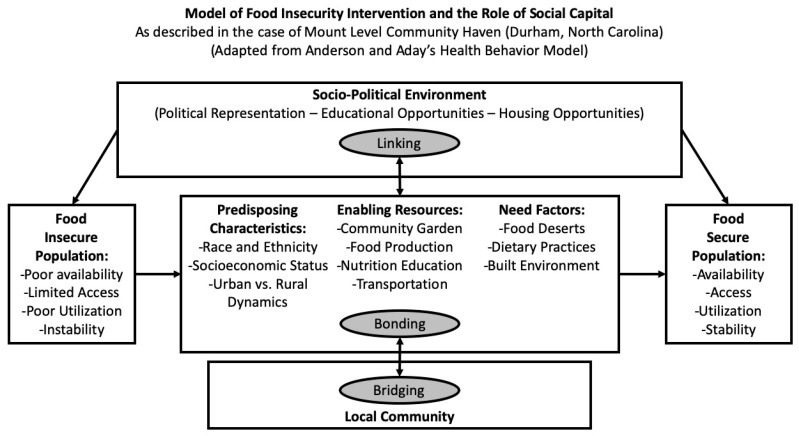
Health behavior model of a food insecurity intervention showing the role of three types of social capital promoting food security.

The potential relationships of social capital to food security in the case of the Mount Level Community Haven (MLCH) can be considered in a health intervention framework as presented in Figure 1. This framework is based upon Anderson’s Behavioral Model of Health Services Use, in which “enabling resources” are leveraged to address the identified needs of a community [4].

### 2.3. Food Security Is Ultimately a Local Phenomenon

While global production and distribution dynamics, and weather events or conflict, determine patterns of food insecurity, food security is ultimately a local and household-based phenomenon. Local environments, including the social environment, determine the constraints to food security. Households make substantial resource allocation decisions based on the local conditions, including those of their social network.

Given that government programs and other formal structures for providing food assistance do not sufficiently address the needs in most communities, social capital-based organizations are important for filling the gap. These include both nonprofit community service organizations, such as food pantries, as well as faith communities. 

Churches and other faith communities serve as important sources of social resources for many, particularly those who are vulnerable. In rural areas, churches may be one of few active community organizations. Further, churches particularly engage the elderly, a vulnerable population for food insecurity. Churches increasingly engage in health interventions. Church leadership such as priests and pastors can serve as guides for health and dietary behaviors [36]. The importance of church leadership in influencing health behaviors may be particularly true in traditional African American communities [37].

The role of churches in ensuring food security is rooted in social capital. Churches provide bonding relationships between members and their pastor, which provides primary attention to the well-being of those with strong bonds. Bridging relationships across a more diverse population of attendees allows both for the development of programming to confront social needs (such as food insecurity) and the coordination or pooling of resources across organizations to support assistance to increase food security. Churches provide pathways for linking relationships to gain support and engage with programming from outside organizations, regional church offices, and as leverage to political power.

## 3. Case Study: Mount Level Community Haven

The Mount Level Community Haven exemplifies the linkages of social capital in ensuring food security as an illustrative case [38]. A case study approach, and specifically an illustrative case study, provides a grounding or “starting point” in an example to demonstrate the plausibility of the theoretical links proposed [39]. Community Haven, a faith-based grassroots organization in suburban Durham County, North Carolina, USA, provides a variety of community services within and beyond the associated church, especially related to food security. MLCH maintains a large community garden that provides multiple functions to enhance food security, including production of food, education about healthy food, and symbolic promotion of food security and health.

The history of MLCH traces its origins to an African American church founded by freed slaves in 1864. Oral history passed down in the church describes the important role of the church in providing shared social support. The church community was forced to relocate for the construction of a military base in 1942, and the community services of the church have grown in complexity to the formation of the faith-based nonprofit, MLCH [40].

MLCH promotes food security by leveraging social capital in the form of bonding, bridging, and linking connections. MLCH serves members of the church and immediate community by providing access through bonding relationships of families within the larger church community. This is done by directly providing food aid as well as providing meal delivery to those who are homebound, including the elderly, the disabled, and those with small children. MLCH also supports bridging relationships by providing food access to the wider community. Finally, MLCH plays an essential role in linking with other stakeholders and policymakers regarding the issues and promotion of programs for food security.

The potential relationships of social capital to food security in the case of MLCH can be understood through the health intervention framework as presented in Figure 1 [4]. Figure 1 shows the Andersen and Aday model of health behavior that indicates how a food-insecure population moves towards being a food-secure population. Socioeconomic characteristics “predispose” populations to food insecurity. However, within a certain socio-political environment, a proximate community can bond through their predisposing characteristics, resources, and needs, as described above in the relationship of bonding social capital and the utilization dimension of food security. “Enabling resources” in the health behavior model, such as a community garden, are leveraged to address the identified needs for health behavior, here, food security. Through the actions of bonding social capital-enabling resources (such as those identified in the efforts of MLCH), the domains of food security are strengthened. Further, including social capital in this model helps explain the role of the local community through bridging and linking relationships to activate change in the socio-economic and political environment, such as through political representation and educational opportunities. To summarize, Figure 1 shows the progression through health behavior actions from a worse state of food insecurity to a better state of food security, specifically relying on the value generated by social capital. 

## 4. Conclusions

As shown in the theoretical relationship between food security and social capital, and suggested in the case example, enhancing multiple forms of social relationships can strengthen food security. This model provides both a new lens with which to consider social capital as well as a pathway for application, as suggested in the illustrative case of MLCH. Programs that wish to address food insecurity should consider the roles in which social relationships influence the effectiveness of interventions to improve food security. Specifically, the value of this research is the practical use of frameworks in structuring responses to the pressing problem of food security. Community groups who desire to address food security should consider the ways to use close bonding relationships, the possibility of linking relationships among the broader community, and the role of the group in bridging relationships to advance institutional or political action.

This research builds upon a variety of local efforts at North Carolina Central University to address food security in the Durham community while addressing questions of food security more broadly in the framework of social capital. Characteristics of a food-secure community are deeply social, as described above in the illustrative case of MLCH. 

Future work will investigate how faith communities play a role in reducing food security, particularly along the lines of high social capital. The project aspires to apply the theoretical model presented in this paper and the baseline case study to both identify and support interventions as detailed in the health behavior model of MLCH in Figure 1 and the prior section.

Food security, described as the availability, access, utilization, and stability of food for individuals or households, is closely linked to mechanisms of social capital, from the direct sharing of food through bonding relationships, to the bridging influence of relationships on nutritional behavior, and to the linking pathways for communities to get assistance for long-term food security. These relationships suggest pathways for further inquiry and intervention.

## Figures and Tables

**Table 1:** Example effects of social capital on food security.

		Social Capital Dimensions		
		Bonding	Bridging	Linking
**Food Security Dimensions**	**Availability**	Sharing of food supplies within household and with neighbors	Community gardens and food pantries	Processes to receive and distribute food assistance
	**Access**	Pooling of resources and sharing of transportation	Informal aid of food, money, or access to transportation	Enhancement of community-based food assistance programs
	**Utilization**	Household food preparation and choice of meal nutrition	Promotion of favorable social norms and dietary behaviors	Creation and promotion of relevant nutritional messaging and education
	**Stability**	Household planning and redistribution of resources	Neighborhood food sharing and assistance to obtain food	Early warning systems and programs for preventing food shortage
